# Tribute to Peter Richard Almond (1937‐2023)

**DOI:** 10.1002/acm2.14184

**Published:** 2023-10-18

**Authors:** Kenneth R. Hogstrom, Michael D. Mills

**Affiliations:** ^1^ Department of Physics & Astronomy Louisiana State University Baton Rouge Louisiana USA; ^2^ Mary Bird Perkins Cancer Center Baton Rouge Louisiana USA; ^3^ Department of Radiation Physics The University of Texas M. D. Anderson Cancer Center Houston Texas USA; ^4^ James Graham Brown Cancer Center University of Louisville Louisville Kentucky USA

## 1 EARLY YEARS AND EDUCATION

Peter was born on 10 September 1937, in Downton, England, to Stanley Richard and Louise Gladys Moody Almond. His parents got quite a shock when Peter's arrival was accompanied by his identical mirror‐twin brother John. Shortly thereafter, the family moved to Edenbridge in Kent where Stanley was the pastor of its Baptist church. Much of Peter's childhood memories were from World War II, which included periods of rationing, overhead German bombers and V‐1 rockets in route to London, nearby casualties, an evacuation, and a German fighter plane crashing nearby. He recalls the surrounding military establishment in southern England, where his dad served on Sundays as a chaplain conducting Free Church services.

Peter's curiosity was exemplified by his sometimes being a mischievous young boy and not the most stellar of students. Early in his academic career, one headmistress declared that Peter and John were going to “end up nothing but ditch diggers.” John later became a civil engineer, which Peter whimsically supposed “a professional ditch digger.” However, Peter and John's behavior was expected to be fitting of a “Son of the Manse” (preacher's kid).

Peter later discovered the joys of studying, especially in math and physical sciences. Although a “winding road,” this led him to the University of Nottingham in 1955, where he received his BSc, Honors Physics in 1958. With National Service simultaneously being phased out, army service would no longer be required, leaving Peter suddenly unsure of his future. Serendipitously, he found his calling when, unexpectedly, he was given the opportunity for a 1‐year postgraduate Certificate Program in Medical Physics from Bristol University, which he completed in 1959. From there Peter came to the United States, accepting an offer for a 1‐year fellowship in medical physics at The University of Texas at Houston (TX) M. D. Anderson Hospital and Tumor Institute (MDAH), later named M. D. Anderson Cancer Center (MDACC). In 1960, he entered the Department of Physics at nearby Rice University, where he received his MS (1962) and PhD (1964) in Nuclear Physics. There, Peter briefly overlapped with Professor T. W. Bonner (1910‐1961), well known for his work in neutron detection and as director of the lab in which Peter studied, posthumously named the T. W. Bonner Nuclear Laboratory.

In 1965, following a 1‐year postdoctoral fellowship in the Department of Physics at MDAH, Peter accepted a faculty position there, advancing to tenured Professor in 1972. As Head of the department's Radiation Physics Section (1972‐1985), Peter valued his faculty and himself having three jobs: (1) patient care to see in flesh the value of their work, (2) research to advance the science and help not only patients at MDACC but others worldwide, and (3) education to teach current and future medical physicists to best do their job and keep abreast of evolving technology. Peter was a good boss in that he provided his team with challenging problems, resources to solve them, and freedom to produce creative solutions.

In 1985, Peter accepted the position of Vice Chairman of Research and Professor in the Department of Radiation Oncology at the University of Louisville, James Graham Brown Cancer Center, from which he retired in 1998. Subsequently, to be near his family, he returned to Houston, TX, where he was appointed Research Professor (part‐time) in MDACC Department of Radiation Physics.

## 2 RESEARCH CONTRIBUTIONS

Peter contributed to many areas in radiation therapy, but he was most renowned for his work in electron beam therapy, dose calibration, and neutron therapy. His success came not only from his scientific prowess, but his ability to collaborate well with fellow medical physicists in the United States and world to further the practice of medical physics.

Early career he and his team researched ion chamber and thermoluminescent dosimetry for electron beam radiation therapy, using a newly acquired Siemens betatron and later a Therac 20 with scanned electron beams. He worked closely with radiation oncologist Norah duV Tapley, MD, publishing the classical book, *Clinical Applications of the Electron Beam* (1976).^1^ Also, he was a major author of *ICRU Report 21—Radiation Dosimetry: Electrons with Initial Energies Between 1 and 50 MeV* (1972)^2^ and its successor, *ICRU Report 35* (1984).^3^

Mid‐career Peter expanded his electron research group to include faculty Ken Hogstrom, PhD, and postdoctoral fellow Michael Mills, PhD. Together the three published numerous articles on the utilization of electron beam dose measurements, dose output calculations, and CT‐based patient dose calculations for treatment planning, all based on their landmark PMB publication *Electron Beam Dose Calculations* (1981).^4^ Early at MDAH and later at the University of Louisville, he and his students contributed significantly to measuring electron beam energy spectra using gas Cerenkov radiation detectors and magnetic spectrometers. As research professor, Peter reunited with the MDACC electron beam research group, collaborating on its laser‐wakefield‐accelerated electron beam research, coauthoring another journal article by Kainz et al. (2004)^5^; that article received the American Association of Physicists in Medicine (AAPM) Farrington Daniels Award, a single annual award for an outstanding paper on radiation therapy dosimetry, planning, or delivery published in *Medical Physics* for the previous calendar year.

Peter, whose PhD research included neutron scattering, was a natural leader for the neutron physics and dosimetry group supporting ^252^Cf brachytherapy and cyclotron‐based external beam, fast neutron radiotherapy clinical trials being conducted by MDACC. Cyclotron patients were initially treated at the Texas A&M cyclotron and later at the MDACC cyclotron facility. Significant contributions included commissioning fast neutron beams and implementing beam flattening filters. The challenge of modeling the latter for treatment planning dose calculations was solved by Hogstrom et al. (1976),^6^ resulting in Peter sharing the first of his two Farrington Daniels Award. Later, in his role as Director of Cyclotron Unit, Peter collaborated on neutron microdosimetry using Rossi chambers and nuclear track detectors to characterize the high‐LET patient beam.

Peter's most impactful work was the calibration of external radiation beams, particularly his leadership in the publications extending the 1983 AAPM TG‐39 protocol for … *use of parallel plate ionization chambers for dosimetry of electron beams* (1994)^7^ and creating *AAPM's TG‐51 protocol for clinical reference dosimetry of high‐energy photon and electron beams* (1999).^8^

## 3 TEACHING LEGACIES

One of Peter's legacies is his teaching. He loved teaching, and he taught multiple graduate medical physics courses while mentoring approximately 25 MS and PhD students and postdoctoral fellows. Many of these later had successful and highly regarded careers at major medical physics academic centers or progressive community cancer centers.

Peter organized numerous medical physics continuing education (CE) courses, including the 1969 and 1976 AAPM Summer Schools on Dosimetry. He is particularly well known for a CE course in High Energy Electron, x‐ray, and Neutron Dosimetry that he created and taught for 20 years at MDACC. This course was important, as radiotherapy delivery transitioned from Cobalt‐60 to megavoltage accelerators as radiation sources.

## 4 PROFESSIONAL SERVICE

Peter's service to his profession was profound. With AAPM, he served on or chaired multiple committees and task groups, and was president (1971). He also served as chairman (1986) of the American College of Medical Physics (ACMP) and as president (1993) of the Council on Ionizing Radiation Measurement Standards (CIRMS). He served many other professional organizations, which included the National Council of Radiation Protection and Measurements (NCRP), the American Society for Radiation Oncology (ASTRO), and others. As a researcher, he served terms as a member and chair of the National Institute of Health (NIH) Radiation Study Section. His knowledge, experience, demeanor, and leadership were evident wherever he served.

In retirement, Peter contributed to medical physics on a part‐time basis. Highly significant, he served as the inaugural Editor‐in‐Chief of the *Journal of Applied Clinical Medical Physics (JACMP)*, thought to be one of the first online, open‐access journals in medicine, which later became the second of the two AAPM journals. In recognition of being its first editor, each year JACMP selects a single publication to receive the Peter R. Almond Award of Excellence for an outstanding radiation measurement article in the previous year's journal.

## 5 JUNE ALMOND AND FAMILY

In 1959, shortly after arriving in Houston, TX, Peter rented an apartment a couple of miles from MDAH and near a bus stop. The following Saturday, he began searching the nearby area for a Baptist church on foot with no map, no phone, and no directory. After a couple of hours, he located South Main Baptist Church about one mile away. He began attending the next day, and a few weeks later he met June Stockstill from Picayune, MS, who had moved to Houston to work as an elementary school teacher and had also begun attending South Main recently. She had a car and drove Peter to and from church, and the two began dating in March 1960. Interestingly, Peter and June double‐dated with Marilyn Stovall and Glen Redden on a camping trip to Big Bend National Park. (Marilyn Stovall, PhD, also worked at MDAH in the Department of Physics, and both later became internationally known, tenured faculty members there). June and Peter were married in August 1960 and were together for just over 61 years before June's passing from Parkinson's disease in January 2022.

## 6 INTERESTS OUTSIDE MEDICAL PHYSICS

In addition to his love for the church, Peter had many interests outside his daily job. These included sports, fishing, collecting and repairing old clocks, reading, writing, and spending time with his family.

Peter was always active in his church, and his teaching experience began early in life. At the age of 15, his father, in addition to pastoring his local church, had oversight over a small Congregational Chapel in an adjacent village that required him to preach there on Sunday afternoons. Thinking Peter was old enough to teach, he told Peter to take over teaching Sunday School for the school‐aged children. Having received no suggestions, ideas, or materials, Peter grabbed a book, *Through the Bible*, that had full‐color illustrations. This became a life pattern in his teaching. Peter taught a Men's Sunday School class at his local church in Houston, TX, for 15 years, and was still preparing Bible study lessons for a group of senior adults until his final days. Appreciation of Peter was no better exemplified than by a church filled with parishioners, friends, and colleagues at his memorial service.

Peter loved sports and was quite successful in high school in rugby, cricket, cross country, and track and field. His love of running was lifelong. He often ran around Hermann Park adjacent to the Texas Medical Center at lunch, and it was difficult to keep pace with him. With little cricket in Houston, he became a Houston Astros baseball fan, attending regularly. He also shared with his grandson Drew a passion for Houston Dynamo soccer, the two going to games together for years. Drew was also his fishing buddy when the family had a beach house on Bolivar Peninsula in Galveston, TX.

Around the house, Peter loved cooking, particularly Yorkshire pudding and bread. He loved instruments that measured, and his house was filled with many kinds of clocks—grandfather clocks, wall clocks, cuckoo clocks—that he repaired and tended to; every hour there was a wonderful cacophony of chimes and dings.

Peter was a voracious reader, often reading two to three books at a time. Peter loved history, and in addition to his medical physics historical lectures and papers, he wrote two books of interest. The first, *Cobalt Blues: The Story of Leonard Grimmett, the Man Behind the First Cobalt‐60 Unit in the United States* (2014),^9^ features early physics at MDAH and its first Department of Physics chair. The second, *Here, There, and Everywhere* (2021),^10^, ^11^ is a memoir written late in life. It details many items outside Peter's professional career.

## 7 CLOSING REMARKS

Peter was well recognized by his peers for his illustrious career. He is a Fellow of The Institute of Physics (AIP), American College of Radiology (ACR), American College of Medical Physics (ACMP), and American Association of Physicists in Medicine (AAPM), and he is a Caswell Fellow of Council on Ionizing Radiation Measurements and Standards (CIRMS). He received the highest awards of the AAPM, the William D. Coolidge Award given once annually for an eminent career in medical physics, and of the ACMP, the Marvin M. D. Williams Award given once annually for an eminent career in clinical medical physics, both gold medal awards.

Peter's professional life was exemplary. He was scientifically challenged and stimulated, and his contributions and advice helped the careers of many medical physicists and the treatment of many radiotherapy patients. Peter was modest, kind, respected, and enjoyed by his professional colleagues and friends. He led a full life serving a purpose for which he was called and well suited, leaving our world a better place. He will be missed but remembered.

Peter died peacefully at home from pancreatic cancer, the same disease that took his twin brother John 29 years earlier. This was Peter's third bout with cancer. In 1995, shortly after John's death, Peter was diagnosed with colon cancer, which was cured with surgery. He was diagnosed with prostate cancer in mid‐2021, and received hormone therapy, then in mid‐2022, he received radiotherapy at the M. D. Anderson Proton Therapy Center. After sounding the gong for the end of treatment, he traveled with his two children to visit family in England, and upon return, he was diagnosed with pancreatic cancer in September 2022.

**FIGURE 1:**
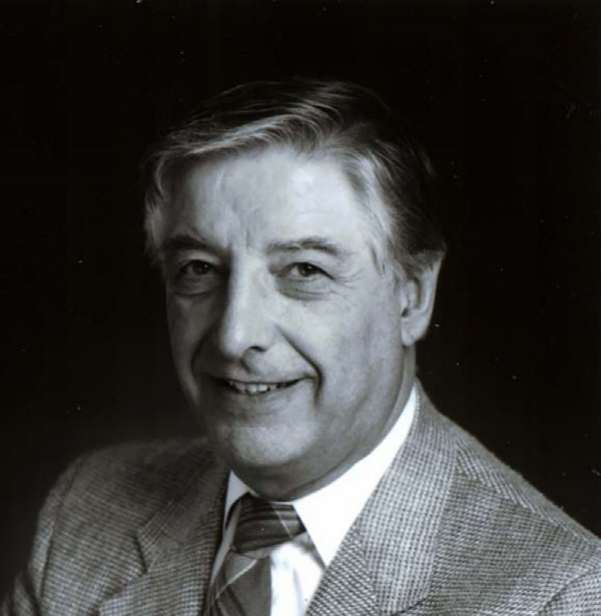
(Portrait, No caption ): (*Source*: The University of Texas MDACC Research Library; https://openworks.mdanderson.org/mchv_interviewsessions/60/).

**FIGURE 2:**
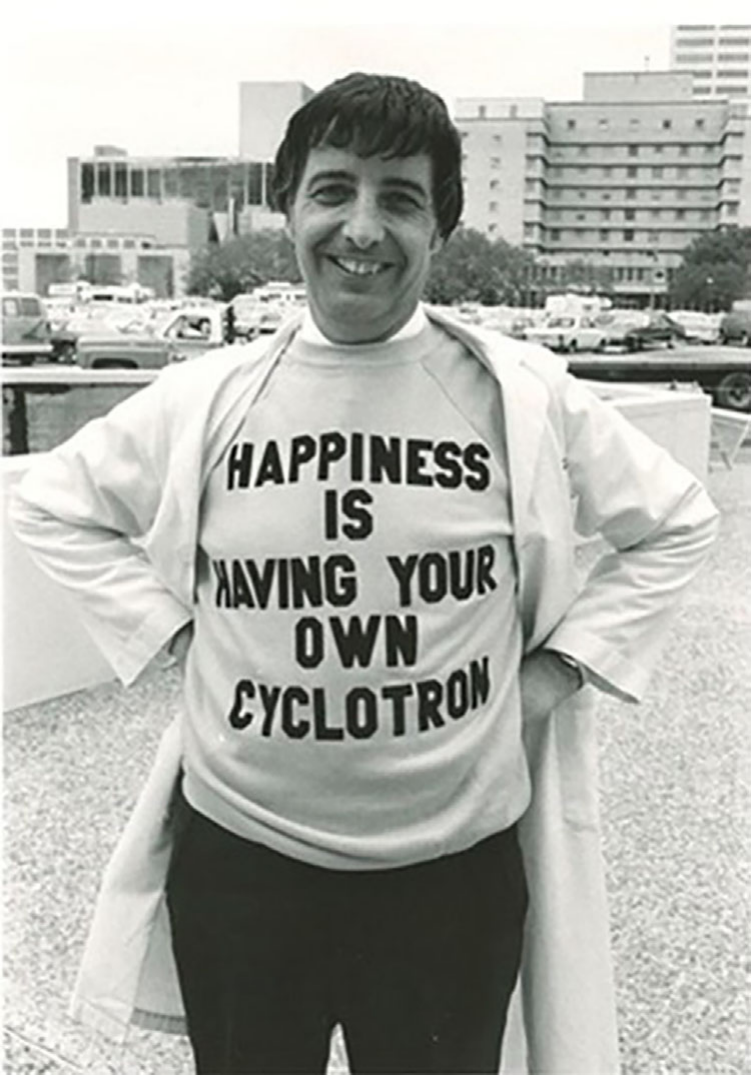
Peter celebrating the delivery of the 42 MeV H^−^ cyclotron from the Cyclotron Corporation as it was being delivered to the basement at MDACC, circa 1982.

**FIGURE 3:**
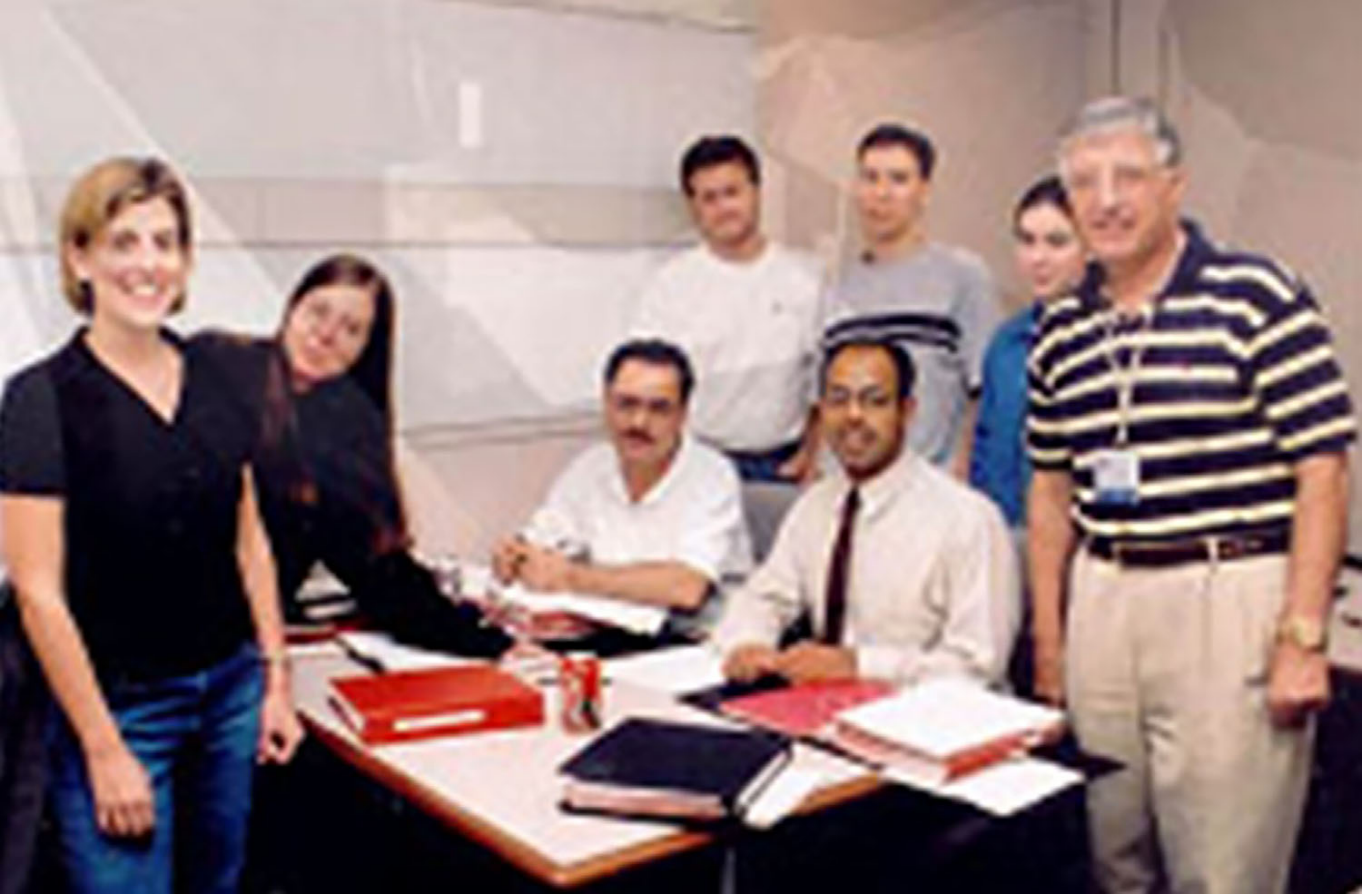
Peter with some of the students in the final offering of the high energy CE short course at MDACC in 2001.

**FIGURE 4:**
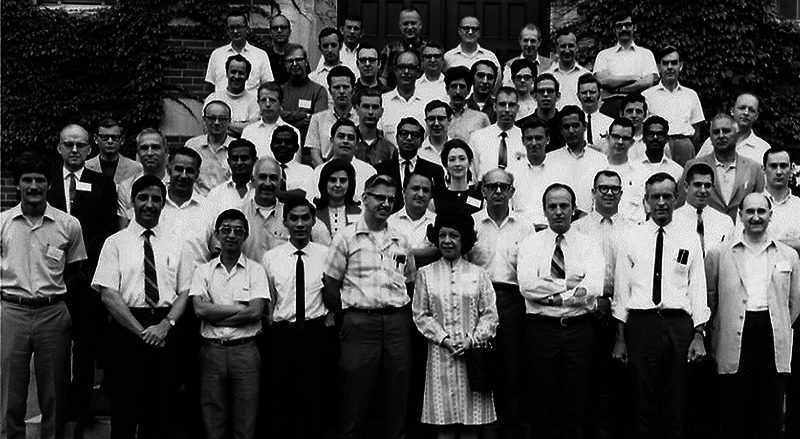
Attendees of the inaugural AAPM Summer School in 1969 on Dosimetry at Burlington, VT, for which Peter (front row, 2nd from left) was director.

**FIGURE 5:**
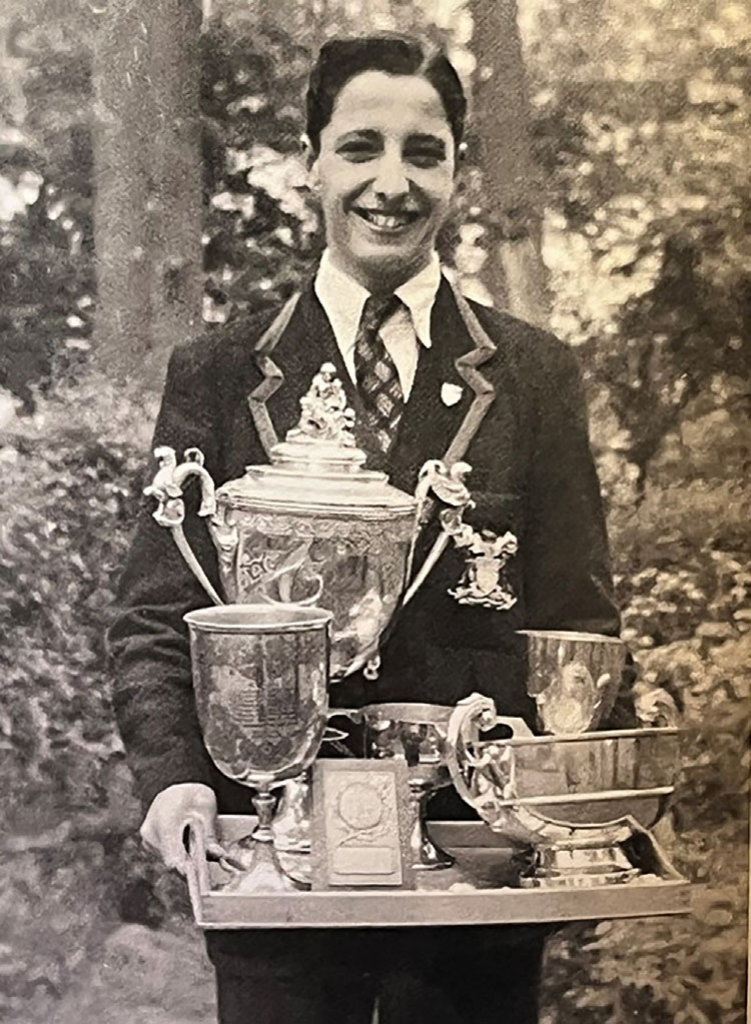
Peter with his trophies while being Victor Ludorum (The Winner of the Games) for winning the most track and field events at Skinners’ School Sports Day, circa 1955 (from Here, There, Everywhere—A Memoir^10^).

**FIGURE 6:**
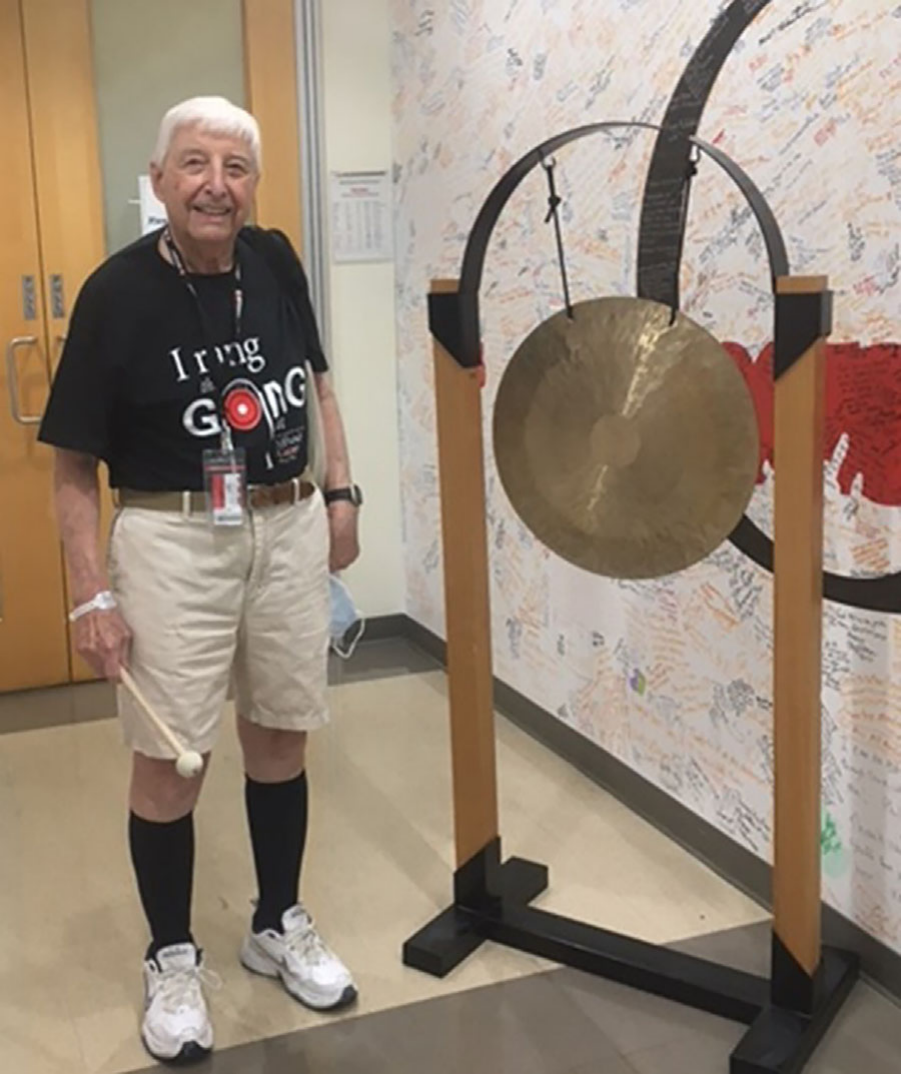
Peter preparing to strike gong signifying the end of his proton treatment at the M. D. Anderson Proton Therapy Facility.

Peter is survived by his son Stephen Almond and wife Christy; daughter Jan Almond Barkley and husband Jim; grandson Drew Barkley and wife Sara; granddaughter Kate Barkley Chopin and husband Lamy IV; sisters Rosemary George and Julia Nicholson and her husband Paul, sister‐in‐law Jan Veranth and husband Frank; and many adored nieces and nephews. He was preceded in death by his wife June Stockstill Almond; his parents Stanley Richard and Louise Gladys Almond; and his twin brother John Downton Almond. Figures 1, 2, 3, 4, 5, 6

## AUTHORS POSTSCRIPT

When considering the life and legacy of Peter R. Almond, we are reminded of this quote by William Barclay^12^: “*There are two great days in a person's life—the day we are born and the day we discover why*.”

Testimonial of Michael Mills: My first class from Peter was not at MD Anderson, but rather I attended his lecture at the South Main Baptist Church on religion, science, the character of the scientist, and the essential nature of virtue in science and clinical practice. In many ways, these were his most important lessons to me. His lectures on Advanced Radiation Physics, while also essential, shared many concepts that were most clearly elucidated in his Sunday School class.

I had many things in common with Peter. Our roots were the M. D. Anderson Cancer Center in Houston. We both served mid‐career as Chief of Physics at the University of Louisville Department of Radiation Oncology. We each had a close brother that passed away much too early in life, and we both edited the *JACMP*. As a scientist, Peter taught me that when you consider a problem like the calibration of photon and electron beams, it is essential to return to first principles and check everything with calorimetry and Fricke dosimetry. Peter was a master of these almost forgotten absolute dosimetry techniques. The legacy of Peter R. Almond is that of a virtuous life as a scientist and one curious of life's greater mysteries.

Testimonial of Ken Hogstrom: Peter played a major role in my career. He challenged me with clinical problems that required innovative physics research to solve. He passed the baton to me to direct his high energy short course while at University of Louisville. He convinced me to be a candidate for AAPM presidency. He was with me step by step in my early career. Even running around Hermann Park, he would be by my side, soaring at the finish to shower first.

My career also shared much with Peter's. We both received our MS and PhD degrees in Nuclear Physics at Rice University's T. W. Bonner Nuclear Laboratory. We both became aware of Medical Physics after a sudden change in our military obligations. We both served as head of the MDACC Radiation Physics Section (later a Department). We both served as AAPM presidents. We shared many of the same awards. We both treasured bringing new technologies to the clinic, particularly for electron beam therapy. We enjoyed lunch together at Captain Benny's seafood and Goode Company's barbecue and pecan pie, maintaining that tradition until his passing. The greatest thing I shared was Peter's legacy of reinforcing the rewards of integrating patient care, research, and teaching.
